# Spatial Heterogeneity of Cadmium Effects on *Salvia sclarea* Leaves Revealed by Chlorophyll Fluorescence Imaging Analysis and Laser Ablation Inductively Coupled Plasma Mass Spectrometry

**DOI:** 10.3390/ma12182953

**Published:** 2019-09-12

**Authors:** Michael Moustakas, Anetta Hanć, Anelia Dobrikova, Ilektra Sperdouli, Ioannis-Dimosthenis S. Adamakis, Emilia Apostolova

**Affiliations:** 1 Department of Botany, Aristotle University of Thessaloniki, 54124 Thessaloniki, Greece; 2 Department of Trace Element Analysis by Spectroscopy Method, Faculty of Chemistry, Adam Mickiewicz University, 61 614 Poznań, Poland; anettak@amu.edu.pl; 3 Institute of Biophysics and Biomedical Engineering, Bulgarian Academy of Science, 1113 Sofia, Bulgaria; aneli@obzor.bio21.bas.bg (A.D.); emya@obzor.bio21.bas.bg (E.A.); 4 Institute of Plant Breeding and Genetic Resources, Hellenic Agricultural Organization–Demeter, Thermi, 57001 Thessaloniki, Greece; ilektras@bio.auth.gr; 5 Department of Botany, Faculty of Biology, National and Kapodistrian University of Athens, 157 84 Athens, Greece; iadamaki@biol.uoa.gr

**Keywords:** bioimaging, clary sage, effective quantum yield (Φ*_PSΙΙ_*), non-photochemical quenching (NPQ), photochemical quenching (*q*_p_), photoprotective mechanism, photosynthetic heterogeneity, phytoremediation, reactive oxygen species (ROS), singlet oxygen (^1^O_2_)

## Abstract

In this study, for a first time (according to our knowledge), we couple the methodologies of chlorophyll fluorescence imaging analysis (CF-IA) and laser ablation inductively coupled plasma mass spectrometry (LA-ICP-MS), in order to investigate the effects of cadmium (Cd) accumulation on photosystem II (PSII) photochemistry. We used as plant material *Salvia sclarea* that grew hydroponically with or without (control) 100 μM Cd for five days. The spatial heterogeneity of a decreased effective quantum yield of electron transport (Φ*_PSΙΙ_*) that was observed after exposure to Cd was linked to the spatial pattern of high Cd accumulation. However, the high increase of non-photochemical quenching (NPQ), at the leaf part with the high Cd accumulation, resulted in the decrease of the quantum yield of non-regulated energy loss (Φ*_NO_*) even more than that of control leaves. Thus, *S. sclarea* leaves exposed to 100 μM Cd exhibited lower reactive oxygen species (ROS) production as singlet oxygen (^1^O_2_). In addition, the increased photoprotective heat dissipation (NPQ) in the whole leaf under Cd exposure was sufficient enough to retain the same fraction of open reaction centers (*q*_p_) with control leaves. Our results demonstrated that CF-IA and LA-ICP-MS could be successfully combined to monitor heavy metal effects and plant tolerance mechanisms.

## 1. Introduction

Cadmium (Cd), a non-essential element for plants, is considered to be as one of the most toxic elements for plants because it is not biodegradable in soil and it accumulates in the environment exhibiting toxic effects [1,2,3]. It can appear in the environment at high concentrations, due to several human activities (industrial and agricultural activities, such as mining and smelting of metalliferous ores, electroplating, wastewater irrigation, and abuse of chemical fertilizers) and subsequently becomes toxic to all living organisms [3,4,5,6]. However, some plants have established several mechanisms for Cd detoxification that result in acclimation and tolerance [2,6].

The photosynthetic process has been shown to be very sensitive to Cd action either directly or indirectly [6,7,8,9,10,11,12,13]. Decrease in the photosynthetic efficiency by Cd may result from stomatal closure, a disorder in enzymatic activities of Calvin-Benson cycle, a decrease in the pigment content, and disturbances in the photosynthetic electron transport [2,6,14,15,16,17,18]. The absorbed light energy is converted into chemical energy via photosystem II (PSII) and photosystem I (PSI) that work co-operatively to transfer efficiently photosynthetic electrons from H_2_O to NADP^+^ (forming NADPH) through the formation of a proton gradient that is used to drive ATP synthesis. PSII catalyzes one of the most exciting reactions in nature, the light-driven oxidation of water and liberation of molecular oxygen [19]. PSII eventually provides the electrons required for the conversion of inorganic molecules into the organic molecules and establishes itself as the “engine of life” [20]. Cd-induced inhibition of PSII photochemistry and linear electron transport may also be due to the limited use of ATP and NADPH by the Calvin-Benson cycle [21] and/or influence on the energy transfer between pigment-protein complexes of the photosynthetic apparatus [13,14]. The Cd toxicity has been reported to affect both photosystems [13,22,23], however, PSII was found to be more sensitive and more affected than PSI [10,13,14,15,16,22,24,25,26]. It has also been suggested that the Cd toxicity influences both donor and acceptor sites of PSII [10,16,21,25]. Nevertheless, Cd has been recently regarded to be PSII donor-side inhibitor as it affects the oxygen-evolving complex [13,14,27]. The effects of Cd toxicity differ by the applied concentrations, the period of exposure, and the plant species [11,26,28,29].

In order to understand the ways of distribution and transport of elements in plant tissues, different techniques of elemental imaging are used, starting from microscopic techniques through techniques based on mass spectrometry and ending with synchrotron X-ray-based techniques [30]. However, undoubtedly, techniques based on mass spectrometry, such as LA-ICP-MS offer a range of advantages in high detection limits and high sensitivity for many elements [31,32]. The coupling of a laser with an ICP-MS as a detector, gives the possibility of wide-range of imaging analysis with good spatial resolution, high sensitivity and capability to image/locate many elements in a single analysis of plant samples [33,34], providing unique data that renders LA-ICP-MS a powerful technique in bioimaging.

Chlorophyll fluorescence analysis has been commonly used as a highly sensitive indicator of the photosynthetic efficiency [35,36,37,38,39,40,41,42], but the photosynthetic function is not uniform at the whole leaf, particularly under abiotic stress conditions [43,44]. This renders conventional chlorophyll fluorescence measurements non-typical of the physiological status of the entire leaf [6,45]. This disadvantage overcomes chlorophyll fluorescence imaging analysis (CF-IA) that reveals spatial heterogeneity of the total leaf area [45,46].

We have previously observed in the metallophyte *Noccaea caerulescens* exposed in hydroponic culture to Cd for three and four days, a spatial leaf heterogeneity of the effective quantum yield of electron transport (Φ*_PSΙΙ_*) [6] that was suggested to arise from differences in the distribution of Cd across the leaf as it was observed by LA-ICP-MS analysis [47]. A future research direction was proposed then to combine CF-IA with LA-ICP-MS to evaluate Cd stress on whole leaves in order to verify this suggestion [6].

Plants used for phytoremediation of polluted soils should be highly tolerant and produce a great quantity of biomass in contaminated conditions despite the accumulation of high amounts of heavy metals in their tissues [2,48,49]. In recent years, increasing attention is paid to the aromatic plants as an alternative for conducting environmentally safe and cost-effective phytoremediation, since these species are mainly grown for secondary products and the contamination of the food chain with heavy metals is eliminated [50].

The herbal plant *Salvia sclarea* L. (clary sage, belongs to the family Lamiaceae) that is tolerant to heavy metals has been attributed to the Zn and Cd accumulators, and has the potential for phytoremediation of soils contaminated with heavy metals [51,52]. It has also been discovered that *S. sclarea* accumulated heavy metals through the root system and the distribution of the heavy metals in organs of the clary sage decreases in the following order: Leaves > roots> stems > seeds [51,52]. However, heavy metal accumulation does not influence its development, as well as the quality and quantity of the essential oils, which can be used in the perfumery and cosmetics [50,51,52]. To the best of our knowledge, the effects of Cd action on the photosynthetic apparatus and PSII photochemistry of Salvia leaves, and especially Cd distribution in the leaf area, have not been studied before.

In the present work we tested the hypothesis whether exposure of *Salvia sclarea* plants to Cd will result in spatial leaf heterogeneity of the effective quantum yield of electron transport (Φ*_PSΙΙ_*), and if it does, whether the decreased Φ*_PSΙΙ_* values in the leaf area will correspond to the respective pattern of high Cd accumulation obtained by LA-ICP-MS analysis.

## 2. Materials and Methods

### 2.1. Plant Material and Growth Conditions

Seeds of *Salvia sclarea* L. collected from a field in the Rose Valley region of Bulgaria were kindly provided by Bio Cultures Ltd (Karlovo, Bulgaria), which is focused on growing several types of herbs for the production of essential oils.

Salvia seeds were germinated and grown on soil in a growth room for about a month. When one pair of true leaves fully expanded (height 4–5 cm), the seedlings were transferred to 1-L vessels (two seedlings per vessel) filled with a continuously aerated modified Hoagland nutrient solution composed of 1.5 mM KNO_3_, 1.5 mM Ca(NO_3_)_2_, 0.5 mM NH_4_NO_3_, 0.5 mM MgSO_4_, 0.25 mM KH_2_PO_4_, 50 μM NaFe(III)EDTA, 23 μM H_3_BO_3_, 4.5 μM MnCl_2_, 5 μM ZnSO_4_, 0.2 μM CuSO_4_, and 0.2 μM Na_2_MoO_4_, adjusted to pH 6.0 and changed regularly every three days. The plants were kept under a photon flux density of about 220 μmol m^−2^ s^−1^, 25/20 °C and 14/10 h day/night photoperiod.

### 2.2. Cd Treatment

About 2-month-old uniform plants were selected and subjected to treatment with 0 and 100 μM Cd (applied as 3CdSO_4_ 8H_2_O) for five days. The nutrient solution with or without Cd was renewed every three days.

### 2.3. Chlorophyll Fluorescence Imaging Analysis

An Imaging-PAM Fluorometer M-Series MINI-Version (Walz, Effeltrich, Germany) was used to measure in 15 min dark-adapted leaves of *S. sclarea* plants, grown with 0 (control) or 100 μM Cd for five days, the effects of Cd on PSII function. Five leaves were measured from five different plants with the actinic light intensity of 220 μmol photons m^−2^ s^−1^. In each leaf, 14–16 areas of interest were selected from which chlorophyll fluorescence values were measured. The chlorophyll fluorescence parameters, measured as described in detail previously [53], were the minimum chlorophyll *a* fluorescence in the dark (*F*_o_), the maximum chlorophyll *a* fluorescence in the dark (*F*_m_), the maximum chlorophyll *a* fluorescence in the light (*F*_m_’), and the steady-state photosynthesis in the light (*F*_s_). The minimum chlorophyll *a* fluorescence in the light was computed by the Imaging Win V2.41a software (Heinz Walz GmbH, Effeltrich, Germany) as *F*_o_*’* = *F*_o_/(*F*_v_/*F*_m_ + *F*_o_/*F*_m_*’*). By using Win software, we calculated the allocation of absorbed light energy at PSII, that is the effective quantum yield of photochemistry (Φ*_PSII_*), the quantum yield of regulated non-photochemical energy loss (Φ*_NPQ_*), and the quantum yield of non-regulated energy loss (Φ*_NO_*). The relative PSII electron transport rate (ETR), the fraction of open PSII reaction centers, the so-called photochemical quenching (*q*_p_) and the non-photochemical quenching that reflects heat dissipation of excitation energy (NPQ) were also calculated.

### 2.4. Laser Ablation Inductively Coupled Plasma Mass Spectrometry

Leaf tissues were analysed in vivo using an ICP-QMS spectrometer (Elan DRC II, Perkin-Elmer Sciex, Guelph, ON, Canada) equipped with a laser ablation system (LA; model LSX-500, CETAC Technologies, Omaha, NE, USA) operating at a wavelength of 266 nm. The instrumentation was optimized on a daily basis by ablating the standard reference glass material NIST SRM 610 and adjusting the nebulizer gas flow, RF generator power and ion lens voltage in order to obtain the maximum signal intensity for ^24^Mg^+^, ^115^In^+^, ^238^U^+^. Plasma robustness was monitored via the ^232^Th^16^O^+^/^232^Th, doubly charged ions and the ^238^U/^232^Th intensity ratios. ThO^+^/Th^+^ intensity ratios were always below 0.2%, doubly charged ions ^42^Ca^2+^/^42^Ca^+^ < 0.5% and ^238^U^+^/^232^Th^+^ intensity ratio was less than 1.2. For leaf sample analysis optimization of the parameters, such as energy of laser beam, spot size, shot frequency and scanning speed, was performed. The laser ablation conditions were chosen so that the ablation of the sample was completed. In the experiment, the volume of standard ablation chamber was reduced to ~10 mL, which shortened the washout time of the aerosol and improved the LA images. The instrumental and analytical conditions of LA-ICP-MS are summarized in Table 1. For bioimage generation, LA-iMageS software was used [54].

### 2.5. Statistical Analysis

Chlorophyll fluorescence analysis data are presented as the mean ± SD. Statistical analysis of means from five leaves from different plants was performed using the Student’s t-test. Differences were considered statistically significant at *p* < 0.05.

## 3. Results

### 3.1. Photosynthetic Heterogeneity Revealed by Chlorophyll Fluorescence Imaging Analysis in Salvia sclarea Leaves under Cd Exposure

**Figure 1:**
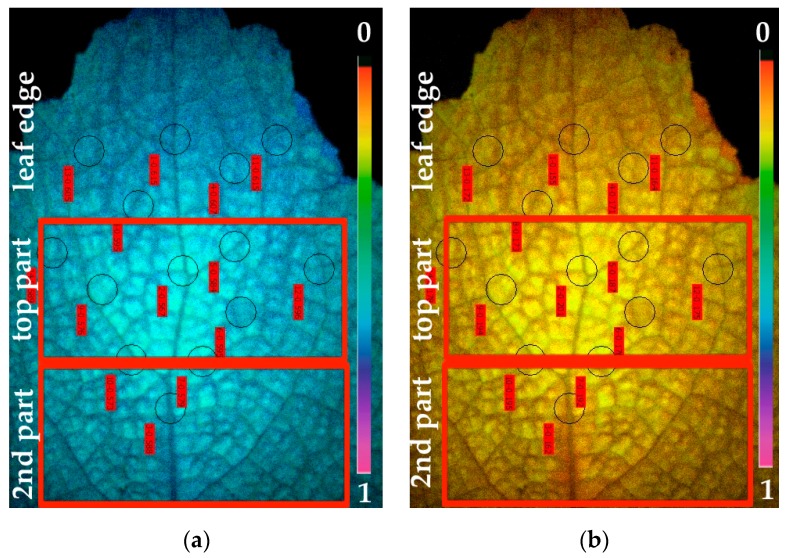
Representative chlorophyll fluorescence images of Φ*_PSΙΙ_* (**a**) and Φ*_NPQ_* (**b**) of *Salvia sclarea* leaves exposed to 100 μM Cd for five days. The different leaf areas: Leaf edge, top leaf part, and second leaf part, are marked. The color code depicted at the right of the images ranges from 0 to 1.

**Figure 2:**
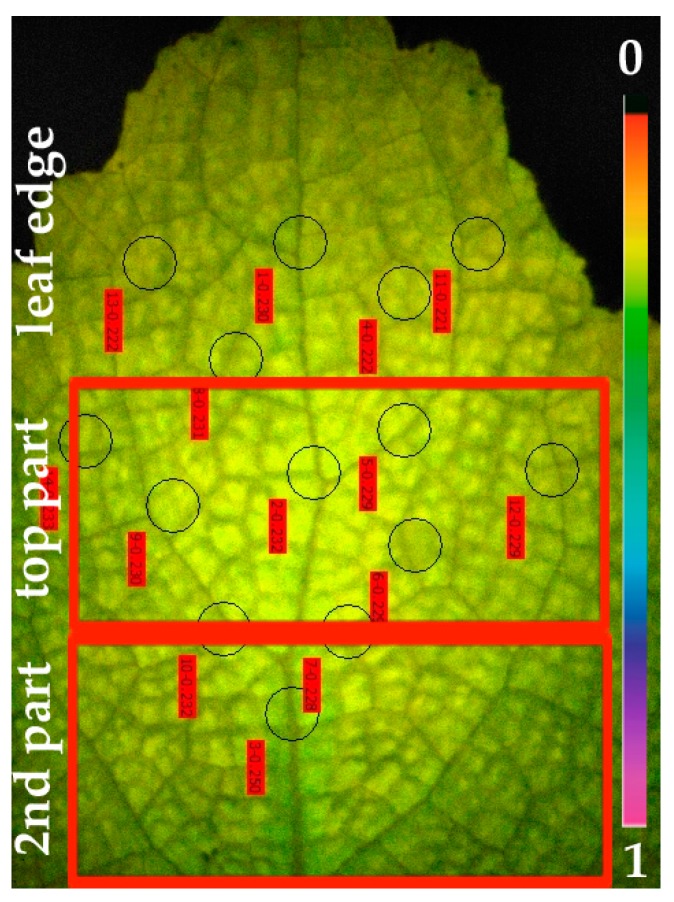
A representative chlorophyll fluorescence image of Φ*_NO_* of *Salvia sclarea* leaves exposed to 100 μM Cd for five days. The different leaf areas: Leaf edge, top leaf part, and second leaf part, are marked. The color code depicted at the right of the image ranges from 0 to 1.

The imaging area in the MINI-Version of the Imaging-PAM Fluorometer M-Series that was used is 24 × 32 mm. Thus, we studied such an area from the distal leaf area of *S. sclarea*. CF-IA revealed a photosynthetic heterogeneity in the studied leaf area of *S. sclarea* leaves under Cd exposure. The spatial heterogeneity was observed mainly in the effective quantum yield of photochemistry (Φ*_PSII_*), the quantum yield of regulated non-photochemical energy loss (Φ*_NPQ_*) and the quantum yield of non-regulated energy loss (Φ*_NO_*) after five days exposure of *S. sclarea* to 100 μM Cd. We observed three clearly distinguishable leaf areas in the chlorophyll fluorescence images of Φ*_PSII_*, Φ*_NPQ_* and Φ*_NO_*. More specifically we marked in the chlorophyll fluorescence images of Φ*_PSΙΙ_*, a leaf area at the leaf edge, than a top leaf area with lower Φ*_PSII_* values than those of the leaf edge, and a second leaf area with Φ*_PSII_* values higher than the top leaf area (Figure 1a). The lower Φ*_PSII_* values, of the top leaf area, were found near the midvein (Figure 1a). The same three areas appeared at the chlorophyll fluorescence images of Φ*_NPQ_* with the top leaf area having higher Φ*_NPQ_* values compared to the other two areas (leaf edge and second leaf area) (Figure 1b). The higher Φ*_NPQ_* values were found near the midvein of the top leaf area (Figure 1b). In the chlorophyll fluorescence images of Φ*_NO_*, the higher values were found in the second leaf area (Figure 2). Representative chlorophyll fluorescence images of Φ*_PSΙΙ_*, Φ*_NPQ_*, and Φ*_NO_* of *Salvia sclarea* leaves from plants grown under control conditions (0 μM Cd) are shown in Figure S1. At control growth conditions, a photosynthetic homogeneity was observed in *S. sclarea* leaves.

### 3.2. Cadmium Imaging in Salvia sclarea Leaves by Laser Ablation Inductively Coupled Plasma Mass Spectrometry

**Figure 3:**
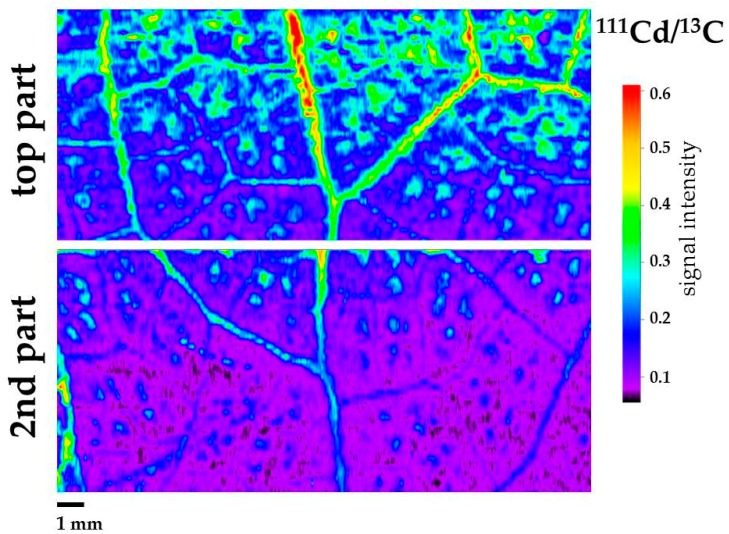
Laser ablation inductively coupled plasma-mass spectrometry (LA-ICP-MS) Cd distribution in a *Salvia sclarea* leaf under Cd exposure (100 μM Cd for five days). The two-leaf areas (marked in Figure 1 and Figure 2) top leaf part and second leaf part, are shown. Cd intensity was normalized using ^13^C.

Three leaves from three different plants were studied by LA-ICP-MS. The area that was selected for analysis corresponds to the two areas that were marked in CF-IA, a top leaf part and a second leaf part. Thus, from each leaf, the corresponding area of 20 × 18 mm was cut and placed on the polyethylene terephthalate slide. In order to normalize the signal, compensating plasma variations and ablations process, two candidates for internal standards, such as ^13^C and ^34^S were evaluated [33]. Finally, isotope of carbon ^13^C was selected as the internal standard as its distribution in the *S. sclarea* leaves was homogeneous. The leaves from *S. sclarea* plants grown under control conditions (0 μM Cd) were analyzed by the whole area (Figure S2), while the leaves from plants exposed to Cd were analyzed in two parts that corresponded to the two leaf parts studied by CF-IA (Figure 3).

The laser beam scanned a selected area of the sample line by line from left to right side with 3 s delay at the end of each line. Approximately 60 lines per leaf were analysed. The number and width of the ablation lines were the same for all leaf samples. Leaves were ablated from the adaxial (upper) side.

The area with high Cd signal intensity was the top leaf area, and, more specifically, the area in the midvein of the top leaf area (Figure 3). In contrast, the presence of Cd was not revealed in leaves of control plants (Figure S2).

### 3.3. Changes in the Light Energy Use at PSII Under Cd Exposure

**Figure 4:**
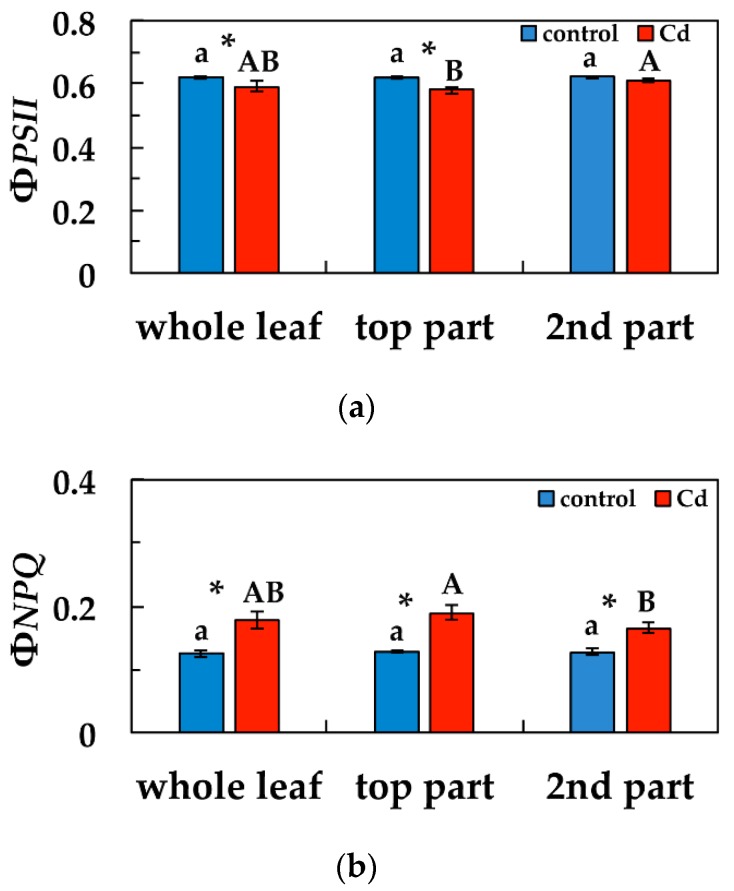
Changes in the quantum efficiency of photosystem II (PSII) photochemistry (Φ*_PSΙΙ_*) (**a**) and the quantum yield of regulated non-photochemical energy loss (Φ*_NPQ_*) (**b**). Whole leaf values, top leaf part area values and second leaf part area values are shown in *Salvia sclarea* plants grown at 0 (control), or 100 μM Cd for five days). Error bars on columns are standard deviations based on five leaves from different plants. Columns with a different letter (lower case for controls and capitals for 100 μM Cd) are statistically different between different leaf areas (*p* < 0.05). An asterisk represents a significantly different mean between controls and 100 μM Cd of the same leaf area (*p* < 0.05).

We calculated for all chlorophyll fluorescence parameters whole leaf values, top leaf part area values and second leaf part area values (leaf part areas are marked in Figure 1 and Figure 2 and Figure S1). We estimated the allocation of absorbed light energy at PSII, that is the effective quantum yield of photochemistry (Φ*_PSII_*), the quantum yield of regulated non- photochemical energy loss (Φ*_NPQ_*), and the quantum yield of non-regulated energy loss (Φ*_NO_*) of *S. sclarea* leaves from plants exposure to 0 and 100 μM Cd. Φ*_PSΙΙ_* whole leaf values, after five days exposure to Cd, decreased significantly compared to controls as did also top leaf part area values compared to their corresponding controls (Figure 4a). Φ*_PSΙΙ_* values of the second leaf part area did not differ compared to the corresponding control values (Figure 4a). The second leaf part values after five days exposure to Cd were significantly higher than the top leaf part area Φ*_PSΙΙ_* values (Figure 4a). Φ*_NPQ_* values after five days exposure to Cd, increased significantly in the whole leaf compared to control, and also in the other two parts compared to their corresponding controls (Figure 4b). Top leaf part Φ*_NPQ_* values after Cd exposure were significantly higher than second leaf part area values (Figure 4b).

**Figure 5:**
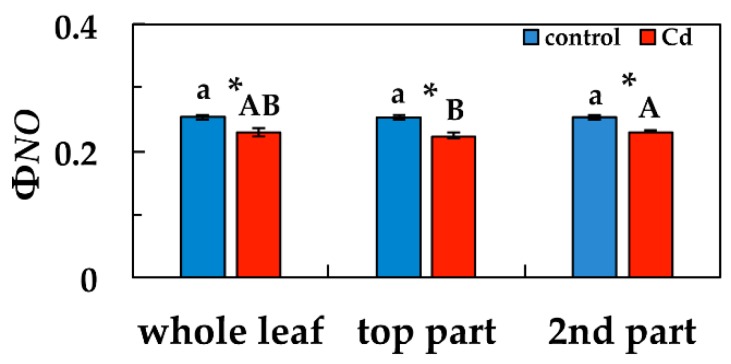
Changes in the quantum yield of non-regulated energy loss (Φ*_NO_*) in *Salvia sclarea* plants grown at 0 (control), or 100 μM Cd for five days. Error bars on columns are standard deviations based on five leaves from different plants. Error bars on columns are standard deviations based on five leaves from different plants. Columns with a different letter (lower case for controls and capitals for 100 μM Cd) are statistically different between different leaf areas (*p* < 0.05). An asterisk represents a significantly different mean between controls and 100 μM Cd of the same leaf area (*p* < 0.05).

Φ*_NO_* values after five days exposure to Cd, decreased significantly in the whole leaf compared to control, and also in the other two parts compared to their corresponding controls (Figure 5). Top leaf part Φ*_NO_* values after five days exposure to Cd were significantly lower than second leaf part area values (Figure 5).

### 3.4. Changes in Non-Photochemical Quenching and the Redox State of PSII under Cd Exposure

**Figure 6:**
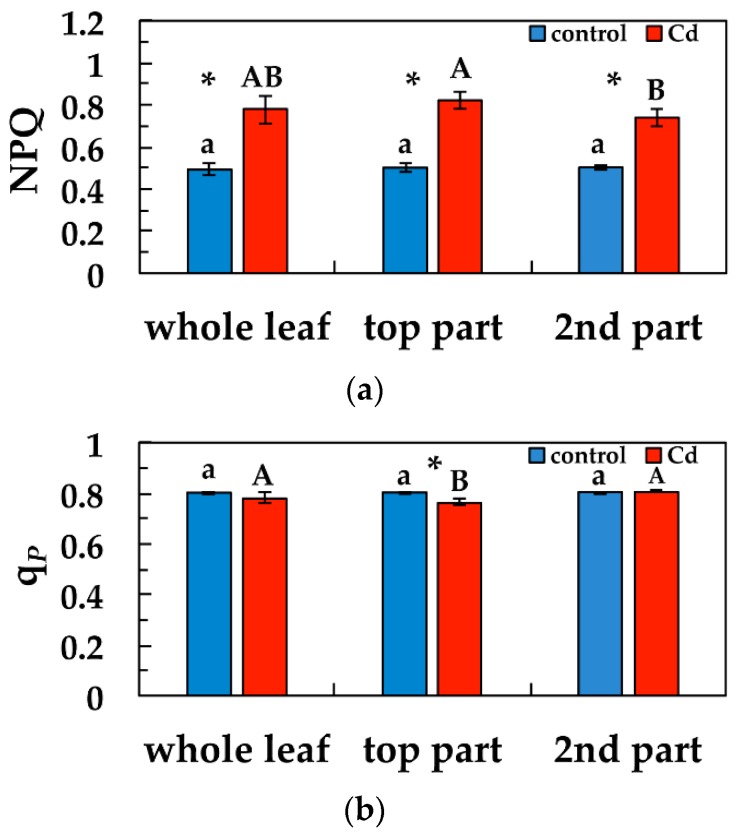
Changes in the non-photochemical fluorescence quenching (NPQ) (**a**) and in the relative reduction state of Q*_A_*, reflecting the fraction of open PSII reaction centers (*q*_p_) (**b**) in *Salvia sclarea* plants grown at 0 (control) or 100 μM Cd for five days. Error bars on columns are standard deviations based on five leaves from different plants. Columns with a different letter (lower case for controls and capitals for 100 μM Cd) are statistically different between different leaf areas (*p* < 0.05). An asterisk represents a significantly different mean between controls and 100 μM Cd of the same leaf area (*p* < 0.05).

Non-photochemical quenching (NPQ) that reflects heat dissipation of excitation energy, increased significantly after five days exposure to Cd in the whole leaf compared to control, and also in the other two parts compared to their corresponding controls (Figure 6a). Top leaf part area NPQ values after five days exposure to Cd were significantly higher than second leaf part area values (Figure 6a). The redox state of PQ pool (*q*_p_), decreased significantly in the top leaf part area compared to the corresponding control, but remained the same with control at the whole leaf level and at the second leaf part area (Figure 6b). Top leaf part *q*_p_ values after five days exposure to Cd were significantly lower than second leaf part values (Figure 6b).

### 3.5. Changes in the Electron Transport Rate in Response to Cd Exposure

**Figure 7:**
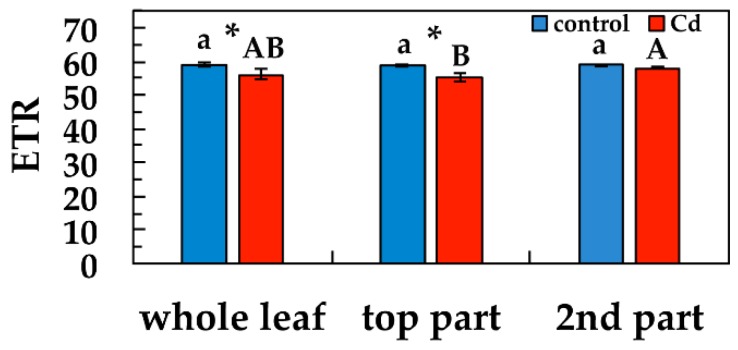
Changes in the relative PSII electron transport rate (ETR) in *Salvia sclarea* plants grown at 0 (control), or 100 μM Cd for five days. Error bars on columns are standard deviations based on five leaves from different plants. Columns with a different letter (lower case for controls and capitals for 100 μM Cd) are statistically different between different leaf areas (*p* < 0.05). An asterisk represents a significantly different mean between controls and 100 μM Cd of the same leaf area (*p* < 0.05).

The relative electron transport rate at PSII (ETR) decreased significantly at the whole leaf level and at the top leaf part, after five days exposure to Cd, compared to their corresponding controls, while retained the same ETR values with controls at the second leaf part (Figure 7). Top leaf part ETR values after five days exposure to Cd were significantly lower than second leaf part values (Figure 7).

## 4. Discussion

Among the different techniques that have been developed for elemental imaging, including secondary ion mass spectrometry, X-ray fluorescence, scanning electron microscopy with energy-dispersive X-ray analysis, and LA-ICP-MS, the latter one has emerged as the prevailing, with high sensitivity and more comprehensible tool for bioimaging of mineral elements in plant tissues [55,56].

Bioimaging of mineral elements in plant tissues has revealed that the distribution of trace elements in leaves is highly heterogeneous [57,58,59]. The accumulation, distribution and localization of Cd in plant leaves, reported by numerous studies [33,47,60], proposed that the accumulation and distribution of Cd and also of other elements depends on the element, the plant species, the organ and the age of the organ [61,62,63]. In Salvia leaves information regarding the distribution of any element is lacking. In our experiment, the distribution of Cd in Salvia leaves, under 100 μM Cd, shows that high Cd signal intensity was detected in the midvein of the top leaf part (Figure 3). In contrast, no Cd could be detected in *S. sclarea* grown under control conditions (Figure S2).

Photosynthetic perturbations to heavy metal exposure do not develop homogeneously over the whole leaf area, thus, making chlorophyll fluorescence measurements at a specific point on the leaf surface non-reliable [43,44]. CF-IA detects spatial and temporal heterogeneity of photochemical efficiency under heavy metal stress and can provide further information on the particular leaf area that is most sensitive to heavy metal stress [6,45].

No significant photosynthetic heterogeneity was detected in leaves of control grown clary sage plants, but we were able to identify a spatial photosynthetic heterogeneity in the leaves of clary sage exposed to Cd. This spatial heterogeneity was observed in Φ*_PSII_*, Φ*_NPQ_* and Φ*_NO_* after exposure to Cd. The lower Φ*_PSII_* values, of the top leaf area, that were found near the midvein (Figure 1a) are linked to the high Cd signal intensity in the midvein of the top leaf area (Figure 3). These observations confirm the previous suggestion [6] that spatial leaf heterogeneity of the effective quantum yield of electron transport (Φ*_PSΙΙ_*) arise from differences in the distribution of Cd across the leaf.

The high increase of Φ*_NPQ_* values after five days exposure to Cd, in the whole leaf area compared to control values, and especially at the top leaf part (Figure 4b), resulted in lower Φ*_NO_* values, compared to control, with the lower values to be observed at the top leaf part (Figure 5). Φ*_NO_* consists of chlorophyll fluorescence internal conversions and intersystem crossing, which leads to the formation of singlet oxygen (^1^O_2_) via the triplet state of chlorophyll (^3^chl^*^) [39,64,65,66]. Consequently, after five days exposure to Cd, ^1^O_2_ decreased in the whole leaf area, compared to control values, and especially at the top leaf part, where the higher Cd signal intensity was scored. This can be explained by the photoprotective mechanism of non-photochemical quenching, that allows for the sequestration of reactive oxygen species (ROS) below critical levels [61]. Otherwise, an increase in ROS triggers remarkable damage to the metabolic machinery, inducing photoinhibition and a generalized damage response [67,68,69].

Non-photochemical chlorophyll fluorescence quenching (NPQ) is a process that takes place in the photosynthetic membranes of plants, algae, and cyanobacteria in which surplus absorbed light energy is dissipated as heat [70,71,72]. This is a molecular adaptation process that represents the fastest response of the photosynthetic membrane to the surplus light energy [70,71]. Thus, the excess light causes rapid saturation of the photosynthetic reaction centers and their eventual closure [69,70]. The excess light energy that cannot be used for photochemistry can damage the most delicate part of the photosynthetic apparatus, the PSII reaction center, which drives the oxidation of water and liberation of molecular oxygen [18]. In order this photodamage to be avoided, the excess excitation energy has to be safely removed by the photoprotective mechanism of NPQ [71,73,74].

The presence of Cd ions increased the heat dissipation of energy as NPQ [75], but this is a photoprotective response mechanism to avoid ROS generation and damage to PSII [71,76,77]. In general, the influence of Cd on photosystems is more serious in PSII than in PSI [78,79]. The photoprotective dissipation of excess light energy (NPQ) under stress conditions can be regarded as efficient only if it is adjusted in such a way to retain the same fraction of open reaction centers as in control conditions [80]. The photoprotective mechanism of NPQ was sufficient in the leaves of clary sage exposed to Cd, since the fraction of open reaction centers at the whole leaf area remained the same to controls (Figure 6b).

Although excess Cd accumulation is harmful to plants [1,2,7], detoxification mechanisms to Cd toxicity that are involved in Cd tolerance and accumulation exist in the hyperaccumulators [6,45]. Plants have developed complicated mechanisms to control concentrations of essential nutrient elements and to diminish the injury from exposure to non-essential metals, but the mechanisms regarding the regulatory network of metal uptake, chelation, transport, sequestration and detoxification which contributes to the alleviation of heavy metal toxicity and photosynthetic tolerance remain to be further elucidated [81,82,83,84].

## 5. Conclusions

Exposure of *S. sclarea* plants to Cd resulted in spatial leaf heterogeneity of Φ*_PSΙΙ_*, with the decreased Φ*_PSΙΙ_* values in the leaf area to correspond to the respective pattern of high Cd accumulation obtained by LA-ICP-MS analysis. We propose that combining the methodologies of chlorophyll fluorescence imaging analysis (CF-IA) and laser ablation inductively coupled plasma mass spectrometry (LA-ICP-MS) can identify the effects of heavy metals on plants and provide information on tolerance mechanisms. We suggest that *S. sclarea* could be characterized as a heavy metal accumulator, as it is tolerant to Cd, and could also potentially be used for phytoremediation.

## Figures and Tables

**Table 1:** Operating conditions for laser ablation inductively coupled plasma mass spectrometry (LA-ICP-MS) system.

**Laser Ablation**	
Instrument	CETAC LSX-500, Nd-YAG
Wavelength [nm]	266
Ablation frequency [Hz]	10
Spot size [µm]	100
Laser energy [mJ]	5.4
Scan rate [µm/s]	80
Distance between scan lines [µm]	20
Scan method	Mapping 2D; scanning
**ICP-MS**	
Instrument	PE Sciex ELAN 6100 DRC II
Nebulizer gas flow [L/min]	1.1
Auxiliary gas flow [L/min]	1.2
Plasma gas flow [L/min]	16
RF Power [W]	1350
Lens setting	Autolens calibrated
Detector mode	Dual (pulse counting and analog mode)
Measured mass to charge ratios	Cd (*m*/*z* 111); C (*m*/*z* 13)
Sweeps	1

## Supplementary Materials

The following are available online at https://www.mdpi.com/1996-1944/12/18/2953/s1, Figure S1: Representative chlorophyll fluorescence images of Φ*_PSΙΙ_*, Φ*_NPQ_* and Φ*_NO_* of *Salvia sclarea* leaves from plants grown under control conditions. Figure S2: LA-ICP-MS Cd distribution of a *Salvia sclarea* leaf under control conditions.
