# Supplementary material for: Spatial Heterogeneity of Cadmium Effects on Salvia sclarea Leaves Revealed by Chlorophyll Fluorescence Imaging Analysis and Laser Ablation Inductively Coupled Plasma Mass Spectrometry
Source: Materials (Basel). 2019 Sep 12;12(18):2953. doi: 10.3390/ma12182953 (PMC6766342; doi:10.3390/ma12182953)
Supplement: Supplementary file 1 [file materials-12-02953-s001.pdf]

materials

Supplementary Information

Spatial Heterogeneity of Cadmium Effects on *Salvia sclarea* Leaves Revealed by Chlorophyll Fluorescence Imaging Analysis and Laser Ablation Inductively Coupled Plasma Mass Spectrometry

Michael Moustakas [1,*], Anetta Hanć [2], Anelia Dobrikova [3], Ilektra Sperdouli [4], Ioannis-Dimosthenis S. Adamakis [5] and Emilia Apostolova [3]

1 Department of Botany, Aristotle University of Thessaloniki, 54124 Thessaloniki, Greece
2 Department of Trace Element Analysis by Spectroscopy Method, Faculty of Chemistry, Adam Mickiewicz University, 61 614 Poznań, Poland
3 Institute of Biophysics and Biomedical Engineering, Bulgarian Academy of Science, 1113 Sofia, Bulgaria
4 Institute of Plant Breeding and Genetic Resources, Hellenic Agricultural Organization–Demeter, Thermi, 57001 Thessaloniki, Greece
5 Department of Botany, Faculty of Biology, National and Kapodistrian University of Athens, 157 84 Athens, Greece
* Correspondence: moustak@bio.auth.gr; Tel.: +30-2310-99-8335

Received: 28 July 2019; Accepted: 09 September 2019; Published: 12 September 2019

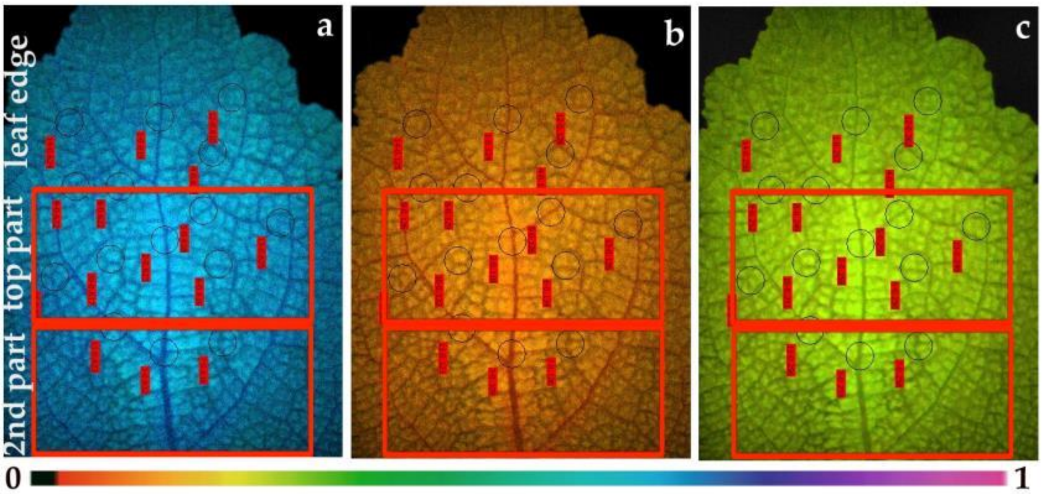

Figure S1. Representative chlorophyll fluorescence images of Φ_{PSII} (**a**), Φ_{NPQ} (**b**) and Φ_{NO} (**c**) of *Salvia. sclarea* leaves from plants grown under control conditions (0 μM Cd). The different leaf areas: leaf edge, top leaf area, and 2nd leaf area, are marked. The color code depicted at the bottom of the images ranges from 0 to 1.

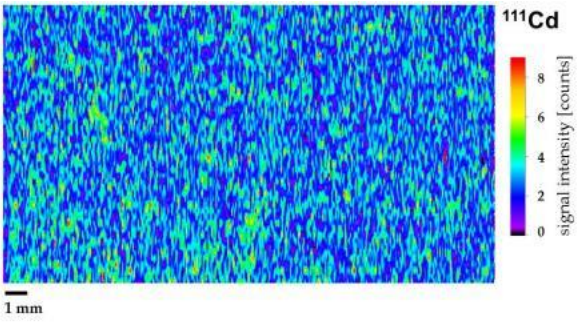

Figure S2. Laser ablation-inductively coupled plasma-mass spectrometry (LA-ICP-MS) Cd distribution of a *Salvia sclarea* leaf under control conditions (0 µM Cd). Image is produced on the raw signal intensity.

© 2019 by the authors. Submitted for possible open access publication under the terms and conditions of the Creative Commons Attribution (CC BY) license (http://creativecommons.org/licenses/by/4.0/).
